# Antibiotic use and adherence to the WHO AWaRe guidelines across 16 hospitals in Zambia: a point prevalence survey

**DOI:** 10.1093/jacamr/dlae170

**Published:** 2024-10-24

**Authors:** Joseph Yamweka Chizimu, Steward Mudenda, Kaunda Yamba, Chileshe Lukwesa, Raphael Chanda, Ruth Nakazwe, Misheck Shawa, Herman Chambaro, Harvey K Kamboyi, Aubrey Chichonyi Kalungia, Duncan Chanda, Sombo Fwoloshi, Elimas Jere, Tiza Mufune, Derick Munkombwe, Peter Lisulo, Tebuho Mateele, Jeewan Thapa, Kenneth Kapolowe, Nyambe Sinyange, Cephas Sialubanje, Nathan Kapata, Mirfin Mpundu, Freddie Masaninga, Khalid Azam, Chie Nakajima, Makomani Siyanga, Nathan Nsubuga Bakyaita, Evelyn Wesangula, Martin Matu, Yasuhiko Suzuki, Roma Chilengi

**Affiliations:** Antimicrobial Resistance Coordinating Committee (AMRCC), Zambia National Public Health Institute (ZNPHI), Lusaka, Zambia; Division of Bioresources, Hokkaido University International Institute for Zoonosis Control, Kita 20 Nishi 10, Kita-ku, Sapporo, Hokkaido 001-0020, Japan; Department of Pharmacy, School of Health Sciences, University of Zambia, Lusaka, Zambia; Antimicrobial Resistance Coordinating Committee (AMRCC), Zambia National Public Health Institute (ZNPHI), Lusaka, Zambia; Action against Antimicrobial Resistance (ReAct) Africa, Lusaka, Zambia; Department of Health, Lusaka District Health Office, Lusaka, Zambia; Department of Pathology and Microbiology, University Teaching Hospitals, Lusaka, Zambia; Department of Pathology and Microbiology, University Teaching Hospitals, Lusaka, Zambia; Hokudai Center for Zoonosis Control in Zambia, Hokkaido University, Lusaka, Zambia; Virology Unit, Central Veterinary Research Institute, Ministry of Fisheries and Livestock, Lusaka, Zambia; Division of Infection and Immunity, International Institute for Zoonosis Control, Hokkaido University, Sapporo, Japan; Department of Pharmacy, School of Health Sciences, University of Zambia, Lusaka, Zambia; Department of Infectious Diseases, University Teaching Hospitals, Lusaka, Zambia; Department of Infectious Diseases, University Teaching Hospitals, Lusaka, Zambia; Department of Post Marketing Surveillance, Zambia Medicines Regulatory Authority, Lusaka, Zambia; Virology Unit, Central Veterinary Research Institute, Ministry of Health, Kabwe District Health Office, Kabwe, Zambia; Department of Pharmacy, School of Health Sciences, University of Zambia, Lusaka, Zambia; Department of Health, World Health Organization, Lusaka, Zambia; Department of Internal Medicine, Levy Mwanawasa University Teaching Hospital, Lusaka, Zambia; Division of Bioresources, Hokkaido University International Institute for Zoonosis Control, Kita 20 Nishi 10, Kita-ku, Sapporo, Hokkaido 001-0020, Japan; Department of Internal Medicine, Levy Mwanawasa University Teaching Hospital, Lusaka, Zambia; Antimicrobial Resistance Coordinating Committee (AMRCC), Zambia National Public Health Institute (ZNPHI), Lusaka, Zambia; Antimicrobial Resistance Coordinating Committee (AMRCC), Zambia National Public Health Institute (ZNPHI), Lusaka, Zambia; Antimicrobial Resistance Coordinating Committee (AMRCC), Zambia National Public Health Institute (ZNPHI), Lusaka, Zambia; Action against Antimicrobial Resistance (ReAct) Africa, Lusaka, Zambia; Department of Health, World Health Organization, Lusaka, Zambia; Strengthening Pandemic Preparedness, Eastern and Southern Africa Health Community, Arusha, Tanzania; Division of Bioresources, Hokkaido University International Institute for Zoonosis Control, Kita 20 Nishi 10, Kita-ku, Sapporo, Hokkaido 001-0020, Japan; International Collaboration Unit, Hokkaido University International Institute for Zoonosis Control, Kita 20 Nishi 10, Kita-ku, Sapporo, Hokkaido 001-0020, Japan; Division of Research Support, Hokkaido University Institute for Vaccine Research and Development, Kita 20 Nishi 10, Kita-ku, Sapporo, Hokkaido 001-0020, Japan; Department of Post Marketing Surveillance, Zambia Medicines Regulatory Authority, Lusaka, Zambia; Department of Health, World Health Organization, Lusaka, Zambia; Strengthening Pandemic Preparedness, Eastern and Southern Africa Health Community, Arusha, Tanzania; Strengthening Pandemic Preparedness, Eastern and Southern Africa Health Community, Arusha, Tanzania; Division of Bioresources, Hokkaido University International Institute for Zoonosis Control, Kita 20 Nishi 10, Kita-ku, Sapporo, Hokkaido 001-0020, Japan; International Collaboration Unit, Hokkaido University International Institute for Zoonosis Control, Kita 20 Nishi 10, Kita-ku, Sapporo, Hokkaido 001-0020, Japan; Division of Research Support, Hokkaido University Institute for Vaccine Research and Development, Kita 20 Nishi 10, Kita-ku, Sapporo, Hokkaido 001-0020, Japan; Antimicrobial Resistance Coordinating Committee (AMRCC), Zambia National Public Health Institute (ZNPHI), Lusaka, Zambia

## Abstract

**Background:**

The inappropriate use of antibiotics in hospitals contributes to the development and spread of antimicrobial resistance (AMR). This study evaluated the prevalence of antibiotic use and adherence to the World Health Organization (WHO) Access, Watch and Reserve (AWaRe) classification of antibiotics across 16 hospitals in Zambia.

**Methods:**

A descriptive, cross-sectional study employing the WHO Point Prevalence Survey (PPS) methodology and WHO AWaRe classification of antibiotics was conducted among inpatients across 16 hospitals in December 2023, Zambia. Data analysis was performed using STATA version 17.0.

**Results:**

Of the 1296 inpatients surveyed in the 16 hospitals, 56% were female, and 54% were aged between 16 and 50 years. The overall prevalence of antibiotic use was 70%. Additionally, 52% of the inpatients received Watch group antibiotics, with ceftriaxone being the most prescribed antibiotic. Slightly below half (48%) of the inpatients received Access group antibiotics. Compliance with the local treatment guidelines was 53%.

**Conclusions:**

This study found a high prevalence of prescribing and use of antibiotics in hospitalized patients across the surveyed hospitals in Zambia. The high use of Watch group antibiotics was above the recommended threshold indicating non-adherence to the WHO AWaRe guidelines for antibiotic use. Hence, there is a need to establish and strengthen antimicrobial stewardship programmes that promote the rational use of antibiotics in hospitals in Zambia.

## Introduction

Antimicrobial resistance (AMR) is a significant global public health threat that leads to increased morbidity and mortality, prolonged illness and higher healthcare costs.^1–4^ This poses a challenge to the treatment and control of infectious diseases.^5–7^ If AMR is not addressed, it could cause more than 10 million human deaths annually by 2050.^2,8–10^ Evidence indicates that AMR occurs naturally, but it is exacerbated by the inappropriate use of antibiotics.^11,12^ Therefore, it is critical to monitor the use of antibiotics in hospitals to develop strategies that promote rational use.^13–16^

The main drivers of AMR include the overuse and misuse of antimicrobial medicines in humans, animals and agriculture.^17^ Antimicrobial stewardship (AMS) programmes are among the critical strategies for reducing the incidence of AMR.^18,19^ AMS programmes promote the appropriate use of antimicrobials that minimize the development of resistance.^20^ These programmes involve optimizing antibiotic prescribing practices, promoting infection prevention and control measures and promoting research and development of new antimicrobial agents.^21,22^ Additionally, AMS programmes provide a forum for interdisciplinary collaboration and continuous quality improvement in antimicrobial prescribing practices.^23–25^ The successful implementation of AMS programmes has been reported to improve the rational use of antibiotics, reduction of antibiotic use and antibiotic-resistant pathogens and a reduction in AMR-related deaths and improve awareness, knowledge, attitudes, perceptions and practices concerning antimicrobial use (AMU) and AMR.^22,26–31^

Previous studies have demonstrated that monitoring antibiotic use in hospitals is critical to promoting rational use.^15,16,32–34^ Some methods established to monitor antibiotic use in hospitals include the World Health Organization (WHO) and Global Point Prevalence Surveys (PPSs).^35–38^ For instance, PPS has been used to collect data on antibiotic use for particular diseases, i.e. community-acquired infections (CAIs) and hospital-acquired infections (HAIs) and monitor adherence to treatment guidelines.^39–41^ In addition, the WHO recommends that of the total medicines prescribed, antibiotics should constitute 40%.^42^ Therefore, PPS can be used to monitor whether prescribing patterns of antibiotics conform to the recommended guidelines. Through PPS, hospitals can determine the appropriateness of antibiotic use among inpatients.^43^ Some studies have reported an increase in the prescription of antibiotics in hospitals.^44–47^ The overuse of antibiotics could be due to disease burden including HAIs.^48^ This increase in antibiotic use could lead to the development and spread of AMR.^49,50^

In 2017, the WHO developed the Access, Watch and Reserve (AWaRe) classification of antibiotics as a tool for AMS and to be used by hospitals to monitor the rational use of antibiotics.^51,52^ The AWaRe classification categorizes antibiotics into the Access group, which comprises narrow-spectrum antibiotics used as a first-line and second-line empiric treatment for common infections.^53,54^ The Watch group comprises broad-spectrum antibiotics with a higher potential of developing resistance and, hence, are used for severe infections, especially among inpatients.^54,55^ The Reserve group are antibiotics used as the last resort and are reserved for treating multidrug resistant infections.^54,56^ The WHO recommends that 60% of the prescribed antibiotics in hospitals must belong to the Access group.^51,54^ Therefore, adherence to the WHO AWaRe classification of antibiotics is vital in promoting rational prescribing and use of antibiotics in hospitals.^52,57^

In Zambia, there are growing concerns about high rates of inappropriate prescribing and use of antibiotics in hospitals reported elsewhere.^58–64^ Furthermore, AMR of microorganisms to commonly used antibiotics has also been reported in hospital settings.^65–74^ There is, however, a dearth of information on the prevalence of antibiotic use in hospitals using PPS methods. Currently, only one PPS has been published, and it was found that the prevalence of antibiotic use was 59%.^64^ A recent study indicated that AMS activities are ongoing in Zambia, and the findings demonstrated a positive impact on awareness, knowledge, attitudes and practices regarding AMU, AMR and AMS.^26^ Additionally, as part of the nationally agreed strategic interventions to this growing concern of AMR, the Government of the Republic of Zambia, with support from cooperating partners, established AMS committees in all the 10 provinces and selected hospitals to promote the prudent use of antimicrobials. This aligns with the fourth objective of the Zambia Multisectoral National Action Plan (NAP) on AMR, which states as follows: ‘To optimize the use of antimicrobial medicines in human and animal health’.^75^ In this regard, the Antimicrobial Resistance Coordinating Committee conducted PPS and later established AMS committees in 16 selected priority hospitals across Zambia. Therefore, this study evaluated the prevalence of antibiotic use and adherence to the WHO AWaRe classification of antibiotics across 16 hospitals in Zambia using a WHO PPS approach.

## Materials and methods

### Study design, sites and population

**Figure 1.**
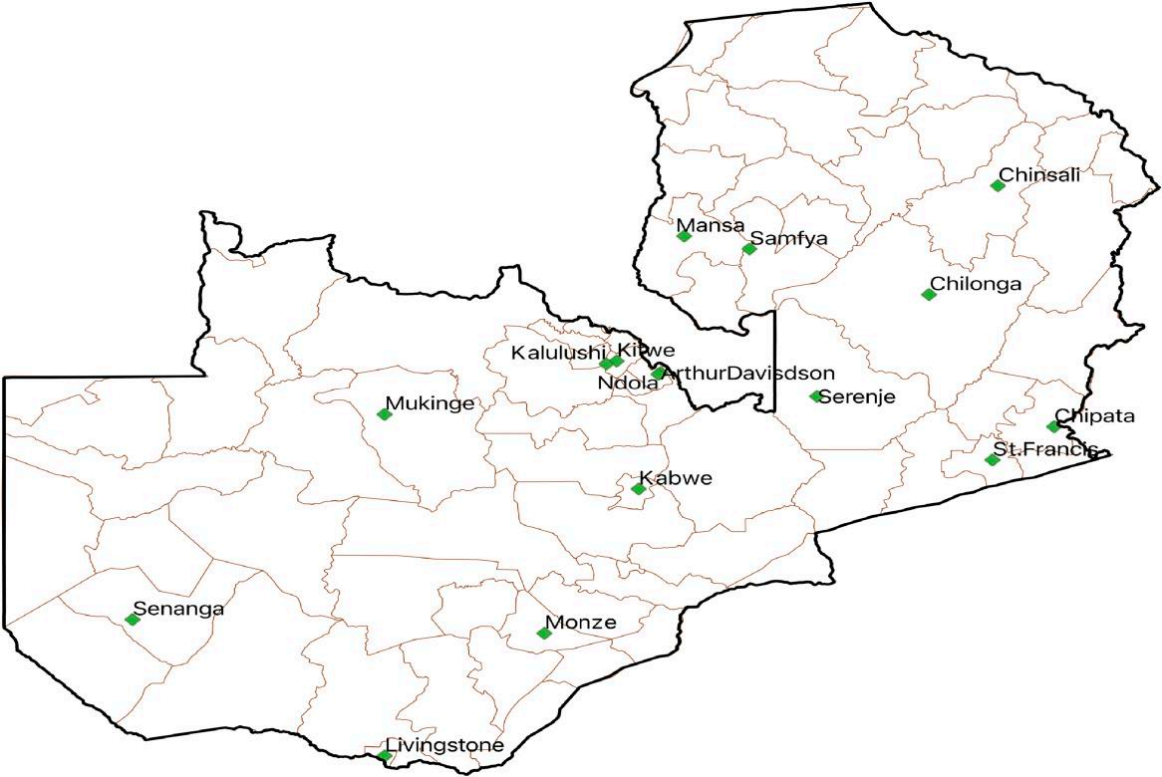
Hospitals included in the Zambia’s PPS, 2023.

This was a cross-sectional study that was conducted in Zambia employing the standard WHO PPS methodology from 11 to 20 December 2023 across 16 hospitals in Zambia. The hospitals were purposively selected because they were in the process of establishing and strengthening AMS programmes in line with the Zambia NAP on AMR. In Zambia, primary, secondary and tertiary hospitals receive referrals from lower facilities, including clinics and health posts. All the surveyed hospitals provide health services to inpatients and outpatients in obstetrics and gynaecology, maternal and child health, infectious diseases, surgery, dental, physiotherapy, pharmacy and radiology. The number of bed spaces ranged from 65 to 741 across the surveyed hospitals. We included all patients admitted to the hospital wards before 08:00 a.m. on the survey day. We included antibiotics for systemic use. We excluded antibiotics for non-systemic use, antiretrovirals, antituberculosis and antifungal medicines. This study excluded outpatients and daytime admissions for ambulatory patients and inpatients on topical antibiotics. The study sites comprised 16 hospitals (6 tertiary-level, 5 secondary-level and 5 primary-level hospitals) as shown in Figure 1 and Table 1.

**Table 1.** Profiles of the surveyed hospitals

Hospital name	Hospital name abbreviations	Province	Hospital type	Hospital ownership	Hospital total beds	Hospital annual admissions
Kalulushi District	KLDH	Copperbelt	Primary	Government	164	8880
Mukinge Mission	MkDH	Northwestern		Faith based	220	12 648
Micheal Sata District	MSH	Muchinga		Government	65	2679
Serenje District	SjDH	Central		Government	81	3693
Samfya District	SmfDH	Luapula		Government	152	5794
Senanga District	SngDH	Western		Government	107	2902
Chilonga Mission	CMGH	Muchinga	Secondary	Faith based	230	3500
Mansa General	MGH	Luapula		Government	420	4908
Monze Mission	MMH	Southern		Faith ased	260	1226
St. Francis Mission	StFMH	Eastern		Faith based	490	15 600
Arthur Davidson’s	ADTH	Copperbelt	Tertiary	Government	260	8500
Chipata Teaching	CTH	Eastern		Government	600	11 200
Kabwe Central	KCH	Central		Government	474	14 940
Kitwe Teaching	KTH	Copperbelt		Government	500	6780
Livingstone Teaching	LTH	Southern		Government	325	10 871
Ndola Teaching	NTH	Copperbelt		Government	741	19 656

### Study population, sample size estimation and sampling technique

The study population included all inpatients who were admitted before 8:00 a.m. on the day of the PPS. A total enumeration sampling technique was employed and included all the inpatients who met the inclusion criteria on the day of the survey. This is in accordance with the WHO PPS methodology, which recommends total enumeration for hospitals with inpatient bed capacity below 500.^42^ We excluded all inpatients with incomplete information in their medical files.

### Data collection

Data collection was done using REDCap version 9.1.15.^76^ We adopted a WHO PPS tool as reported in other studies.^36,42,77^ A training of trainers on how to conduct a PPS was held and composed of selected healthcare workers (clinicians, pharmacists, laboratory officers, nurses and public health officers) from the targeted hospitals who later trained more hospital staff with support from the National facilitators. Therefore, before data collection, 240 multidisciplinary healthcare workers (48 clinicians, 48 pharmacists, 48 nurses, 48 laboratory scientists and 48 public health officers) were trained to collect data using the WHO PPS methodology for 2 days per facility. After the training, simulations were performed to ensure that data collectors understood the data collection and entry process.

Additionally, a pilot study was conducted to ensure that the data collectors were competent in the field. An expert facilitator accompanied each group of data collectors to monitor the data collection process. At each study site, the variables of interest included hospital information (type and total bed capacity), inpatient socio-demographics (age, gender), date of admission, diagnosis, antibiotic prescribed/used and compliance with local treatment guidelines.^42^ Based on the WHO PPS methodology, hospital wards were categorized into medical, surgical, paediatric, gynaecology and obstetrics, and mixed wards. Data collection per hospital was done for 2 days.

### Data analysis

The data collected through REDCap version 9.1.15 were exported to Microsoft Excel 2016. Before analysis, the data were cleaned and checked for completeness. Descriptive analyses, including frequencies and percentages, were used to summarize the data. We categorized all the prescribed antibiotics according to the WHO AWaRe classification of antibiotics.^51–53,55,78,79^ We analyzed the appropriateness of antibiotic therapy by assessing compliance with the Zambia National Standard Treatment Guidelines (STGs).^80^ Data analysis was performed using STATA version 17.0.

### Ethical approval

Ethical approval was obtained from the Tropical Diseases Research Centre Ethics Committee with a protocol ID number TRC/C4/09/2023. All data collectors and inpatients or caregivers were informed of the purpose of the study. Approval to collect the PPS data was provided by the Medical Superintendents of each hospital sampled.

## Results

In this PPS, 1296 inpatients were enrolled, of which 56% (*n* = 730) were female, and 54% were aged between 16 and 50 years. Most patients came from the medical and surgical wards. The prevalence of antibiotic use was 70% (Table 2).

**Table 2.** Demographic and clinical characteristics of patients in Zambia’s PPS, 2023 (*N* = 1296)

Variable	Attribute	*n*	Percentage
Gender	Female	730	56
	Male	566	44
Age range (years)	0–15	358	27
	16–50	696	54
	Above 50	242	19
Hospital	ADTH	97	7
	CMGH	22	2
	CTH	162	13
	KCH	147	11
	KLDH	31	2
	KTH	99	8
	LTH	73	6
	MGH	153	12
	MkDH	88	7
	MMH	92	7
	MSH	18	1
	NTH	146	11
	SjDH	33	3
	SmfDH	26	2
	SngDH	18	1
	StFMH	91	7
Ward	Maternal	239	18
	Medical	319	25
	Mixed ward	56	4
	Other	54	4
	Paediatric	300	23
	Surgical	328	25
Antibiotic use	Patients on antibiotics	908	70

**Figure 2.**
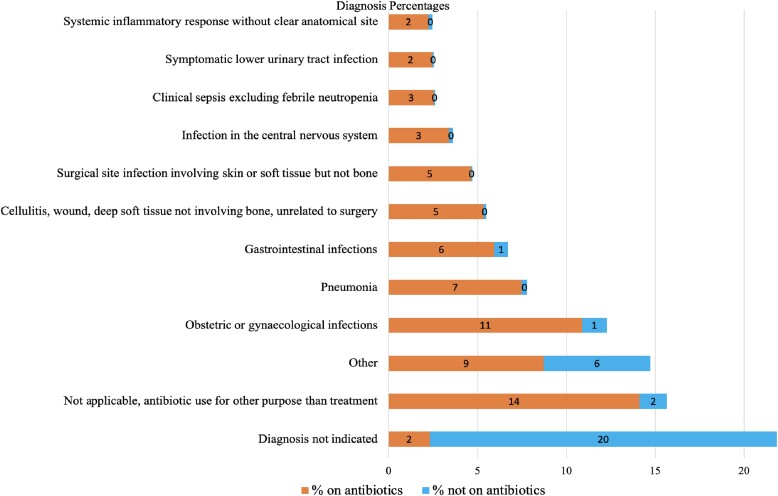
Distribution of antibiotic use per commonly diagnosed infections in the surveyed hospitals. Other includes febrile neutropenia or other infection in the immunocompromised host without clear anatomical site; infections of ear, nose, throat, larynx and mouth, completely undefined, site with no systemic inflammation; intra-abdominal sepsis, including hepatobiliary; cardiovascular infections, endocarditis and vascular graft; and symptomatic upper urinary tract infection.

This study found that most patients were diagnosed with obstetrics and gynaecology infections (12%), pneumonia (7%) and gastrointestinal infections (7%) (Figure 2). The proportions of antibiotic use were higher (14%) in patients where they were used for other conditions than treatment when compared with diagnosis such as obstetric and gynaecological infections (11%) and pneumonia (7%). Of the 432 inpatients who suffered from CAIs, 406 (94%) received antibiotics. Additionally, of the 57 patients who had HAIs, 52 (91%) received antibiotics. This study found that most inpatients received antibiotics for surgical and medical prophylaxis. Of the 186 patients who had surgeries, 180 (97%) received antibiotics for prophylaxis. Additionally, of the 92 patients with medical conditions, 88 (96%) received antibiotics for prophylaxis.

**Figure 3.**
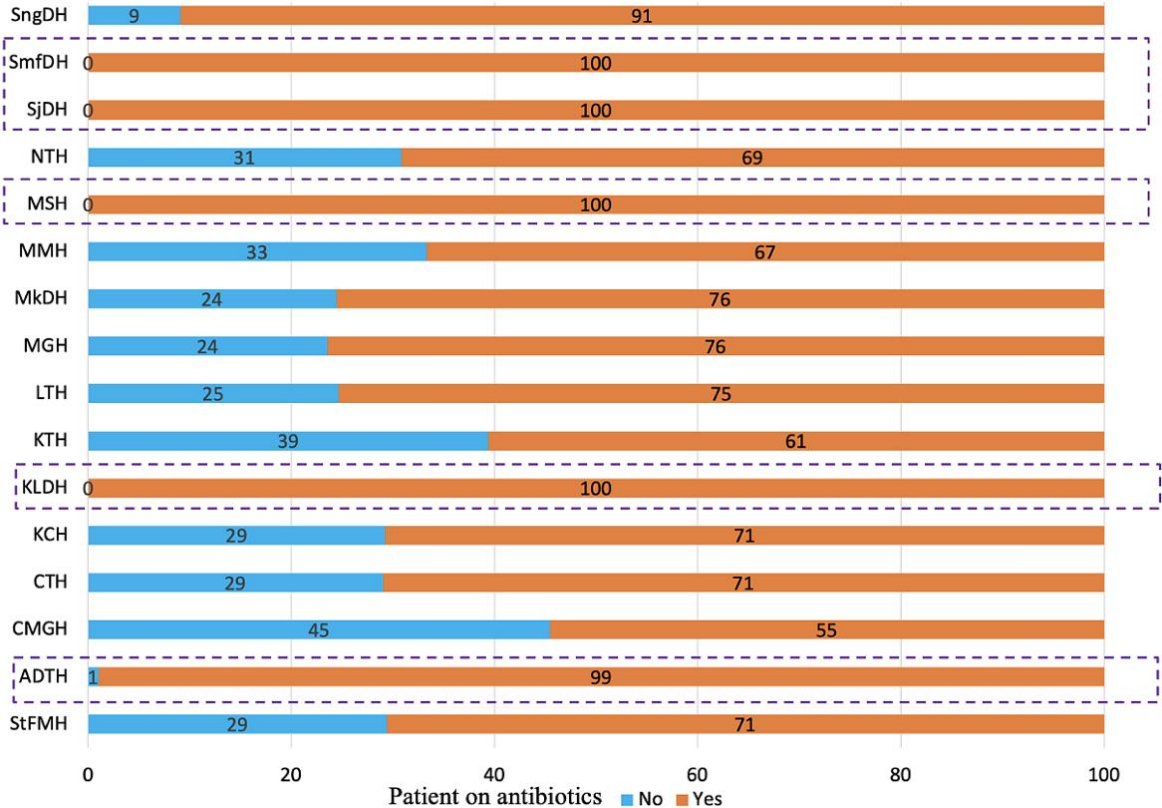
The proportion of inpatients was treated with antibiotics across the surveyed hospitals.

This study found that the percentage of antibiotic use ranged from 55% to 100% across the surveyed hospitals (Figure 3). The use of antibiotics was 100% in four hospitals including SmfDH, Serenje District Hospital (SjDH), Michael Sata District Hospital (MSH) and KLDH (Figure 3).

This study found that of the 899 inpatients on antibiotics, 434 (48%) were on the Access group of antibiotics, while the remaining 465 (52%) were prescribed with the Watch group (Table 3).

**Table 3.** Antibiotic use per hospital according to the WHO AWaRe classification of antibiotics

Antibiotic name	NTH	ADTH	CTH	KCH	KTH	LTH	MGH	MMH	StFMH	CMGH	KLDH	SjDH	SmfDH	MSH	SngDH	MkDH	Total	%
*Ceftriaxone*	58	13	34	38	20	15	69	29	20	3	15	9	16	9	12	21	381	42
**Benzylpenicillin**	5	43	9	10	13	15	6	6	6	0	4	3	2	4	0	0	126	14
**Metronidazole**	13	2	26	23	5	6	8	3	7	1	1	1	3	3	2	8	112	12
**Amoxicillin**	5	2	5	3	6	1	10	1	7	2	2	4	0	0	1	12	61	7
Other	2	2	10	8	3	4	6	4	3	1	0	3	0	0	0	8	54	6
**Cloxacillin**	0	8	6	10	1	1	1	1	5	4	1	1	0	0	0	3	42	5
*Cefotaxime*	0	6	5	0	1	3	2	4	7	0	0	2	0	0	0	1	31	3
*Ciprofloxacin*	0	2	2	3	2	6	2	1	5	0	1	0	0	0	0	3	27	3
*Erythromycin*	1	0	0	8	0	0	2	1	0	0	0	6	1	0	1	3	23	2
**Cefalexin**	1	1	4	0	1	1	1	4	0	0	0	0	0	0	0	2	15	2
**Gentamicin**	0	2	1	0	1	0	3	0	1	0	2	1	2	1	0	0	14	2
**Benzathine benzylpenicillin**	1	0	1	0	0	0	4	2	0	0	3	0	0	0	0	1	12	1
*Azithromycin*	6	0	2	0	1	1	0	0	0	0	0	0	0	0	0	0	10	1
Total	92	81	105	103	54	53	114	56	61	11	29	30	24	17	16	62	98	100

Other includes cefepime (*n* = 5), doxycycline (*n* = 9), nitrofurantoin (*n* = 7), ampicillin (*n* = 2), arbekacin (*n* = 1), benzathine phenoxymethylpenicillin (*n* = 2), ceftizoxime (*n* = 2), levofloxacin (*n* = 1), meropenem (*n* = 5), penamecillin (*n* = 1), phenoxymethylpenicillin (*n* = 2), sulfamethoxazole (*n* = 6) and trimethoprim (*n* = 9). Bold: access group antibiotics; italic: watch group antibiotics.

**Figure 4.**
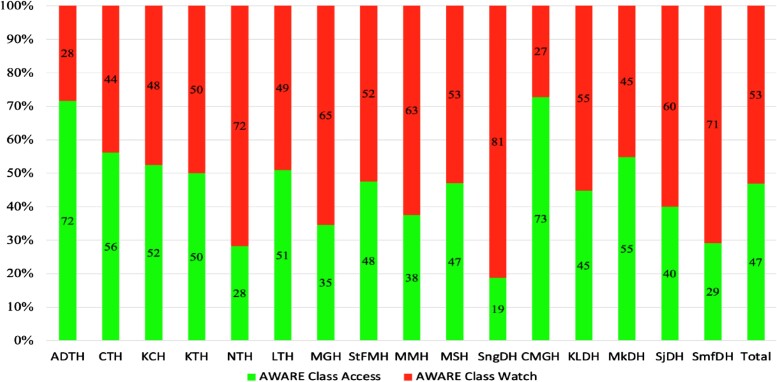
Antibiotic prescribing patterns by the WHO AWaRe classification of antibiotics per surveyed hospital in Zambia.

This study found that 15 of the 16 hospitals had Access group antibiotics prescribed below 60% with only one hospital prescribed 73% of Access group antibiotics for hospitalized patients (Figure 4).

This study found that the overall compliance with the Zambian STGs was 53%, with the lowest being 0% in two hospitals (Michael Chilufya Sata District Hospital and Senanga District Hospital) and the highest being 57% in two hospitals (Chipata Teaching Hospital and Mansa General Hospital) (Table 4).

**Table 4.** Compliance with the Zambian Standard Treatment Guidelines

Facility level	Hospital ID	Compliance with STGs Yes, *n* (%)
Tertiary	Arthur Davidsons Children’s Hospital	43 (53)
	Chipata Teaching Hospital	57 (54)
	Ndola Teaching Hospital	55 (60)
	Kabwe Central Hospital	47 (45)
	Kitwe Teaching Hospital	27 (50)
	Livingstone Teaching Hospital	22 (42)
Secondary	Mansa General hospital	57 (50)
	Monze Mission Hospital	37 (66)
	St. Francis Mission Hospital	34 (56)
	Michael Chilufya Sata District Hospital	0 (0)
	Chilonga Mission General Hospital	4 (36)
Primary	Serenje District Hospital	17 (57)
	Samfya District Hospital	21 (88)
	Senanga District Hospital	0 (0)
	Kalulushi District Hospital	13 (45)
	Mukinge District Hospital	46 (74)
	Overall compliance with STGs	480/908 (53)

The use of antibiotics was significantly higher in patients aged 15 years and below, as well as in those primary hospitals (*P* < 0.001) (Table 5). Multivariate analyses revealed no statistically significant difference in antibiotic use associated with ward type, HIV and malnutrition status, CAI, HAI, surgical and medical prophylaxis (Table 6).

**Table 5.** Associations between antibiotic use and characteristics assessed in this study

			Univariate model				Multivariate model			
Variable (*N*)		Antibiotic use, *n* (%)	OR	95% CI		*P* value	OR	95% CI		*P* value
Gender	Male (542)	417 (77)	1.287	0.997	1.668	0.054				
	Female (729)	526 (72)	1							
Age	≤15 (387)	325 (84)	1							
	≥16 (909)	619 (68)	0.41	0.298	0.558	0.001	0.391	0.199	0.744	0.005
Hospital level	Primary (213)	183 (86)	1							
	Secondary (358)	248 (69)	0.307	0.182	0.498	<0.001	0.342	0.197	0.581	0.001
	Tertiary (725)	519 (72)	0.313	0.191	0.492	<0.001	0.404	0.242	0.659	0.001
Wards	Maternal (239)	165 (69)	1							
	Medical (319)	219 (69)	1.003	0.693	1.446	0.989				
	Mixed (56)	45 (80)	2.489	1.173	5.936	0.026				
	Other (54)	39 (72)	1.201	0.625	2.418	0.593				
	Paediatric (300)	249 (83)	2.395	1.582	3.657	<0.001				
	Surgical (328)	233 (71)	1.108	0.768	1.597	0.581				
HIV status	No (565)	408 (72)	1							
	Unknown (562)	407 (72)	1.129	0.864	1.476	0.374				
	Yes (169)	135 (80)	1.532	1.018	2.356	0.046				
Malaria status	No (1165)	846 (73)	1							
	Yes (131)	104 (79)	1.382	0.9	2.191	0.153				
Malnutrition status	No (595)	460 (77)	1							
	Unknown (592)	393 (66)	0.654	0.503	0.85	0.002				
	Yes (109)	90 (83)	1.38	0.828	2.407	0.235				
CAI	No (863)	537 (63)	1							
	Yes (433)	406 (94)	8.782	5.875	13.675	<0.001				

Statistical significance is denoted by *p* values less than 0.05.

**Table 6.** Associations between antibiotic use and type of indication

			Univariate model				Multivariate model			
Variable (*N*)		Antibiotic use, *n* (%)	OR	95% CI		*P* value	OR	95% CI		*P* value
	Yes (433)	406 (94)	8.782	5.875	13.675	<0.001				
HAI	No (1239)	898 (72)	1							
	Yes (57)	52 (91)	3.77	1.643	10.907	0.005				
Surgical Prophylaxis	No (1110)	770 (69)	1							
	Yes (186)	180 (97)	12.661	6.064	32.429	<0.001				
Medical prophylaxis	No (1204)	862 (72)	1							
	Yes (92)	88 (96)	8.337	3.446	27.437	<0.001				

Statistical significance is denoted by *p* values less than 0.05. The multivariate model included age range, ward type, indication [cCAIs (HAI), hospital-HAIs, surgical and medical prophylaxis], HIV status and malnutrition status.

## Discussion

This is the first PPS on antibiotic use to be conducted across many hospitals in Zambia. Our study found a high use of antibiotics across the surveyed hospitals with a low (53%) compliance with the Zambian STGs. Notably, ceftriaxone, a Watch group antibiotic and third-generation cephalosporin (3GC), was the most prescribed antibiotic.

The present study found that 70% of the inpatients were on antibiotics, indicating a high prescription and use of antibiotics. The reported antibiotic use (ranging from 55% to 100%) across the hospitals is higher than the 40% recommended by the WHO. The high use of antibiotics in hospitals has been reported in other countries in the African region.^81^ In this regard, a PPS conducted in Tanzania reported that 62.3% of inpatients in six referral hospitals in Tanzania received antibiotics.^82^ Likewise, high prescription and use of antibiotics in hospitals were reported in other countries and ranged from 67.7% to 88.2%.^45,47,77,83–89,90–92^ Other PPS reported antibiotic prescription and use that surpassed the WHO recommendations for hospitalized patients and ranged from 43% to 63.8%.^93–100–101^ The prevalence of antibiotic use in hospitalized patients found in our study is higher than what was reported in other studies ranging from 26.14% to 38%.^32,39,40,102–108^ There is a need to conduct PPS in hospitals to monitor the prescribing of antibiotics and the effectiveness of antibiotic policies that influence the implementation of AMS programmes.^109^

The high antibiotic prescriptions in developing countries could be related to the higher infectious disease burden than in Western countries. In the present study, the high use of antibiotics was due to the high prevalence of CAIs and surgical procedures. However, the diagnostic capacity of hospital laboratories in Zambia is limited, and microbiological diagnosis takes long compelling clinicians to prescribe broad-spectrum antibiotics without laboratory evidence.^107,110,111^ Other PPS also found a high use of antibiotics in patients who had CAIs.^38,47,86^ A PPS in Ghana reported a high use of antibiotics in surgical patients.^85^ Another PPS done in Malaysia reported a high use of antibiotics in surgical patients.^112^ Hence, the high burden of diseases has contributed to the overuse and misuse of antibiotics in hospitals.^113^

The present study found that Watch group antibiotics were prescribed in 52% of the inpatients, while Access group antibiotics were prescribed in 48% of encounters. Our findings indicate a deviation from the WHO recommendations of prescribing at least 60% of Access group antibiotics for inpatients.^51,114^ Only Chilonga Mission General Hospital used 73% of Access group antibiotics. The high use of Watch antibiotics has been reported to be common in lower- and upper-middle-income countries.^115^ A PPS done in India found that 53.1% of the prescribed antibiotics were from the Watch group.^89^ Another study conducted across 13 hospitals in Uganda found that 44% of Watch group antibiotics were prescribed for hospitalized patients.^47^ The high use of Watch group antibiotics is happening in many countries.^112,116–120^ This is discouraged by the WHO because these antibiotics are meant to be used for severe infections and have the potential to develop resistance.^51,53,55,56,121^

Conversely, a PPS across 17 hospitals in four countries, including Ghana, Tanzania, Uganda and Zambia, found that most Access group antibiotics were prescribed.^122^ The use of Access group antibiotics in hospitals has been recommended by the WHO and is critical in monitoring the prescribing of antibiotics and promoting their rational use.^123^ There is a need to improve the availability and access to Access group antibiotics to increase their use as recommended by the WHO.^124^ The Zambian PPS study found no Reserve group antibiotics prescribed in all the surveyed hospitals, whereas a PPS in India found that 5.5% of the prescribed antibiotics were from the Reserve group.^89^ Adhering to the WHO AWaRe classification of antibiotics is essential for monitoring antibiotic consumption, defining targets and monitoring the effects of AMS policies that aim to optimize antibiotic use and kerb AMR.^52,114^

Our study shows that ceftriaxone, 3GC, was the most prescribed antibiotic. Ceftriaxone is a Watch group antibiotic and has been reported to be commonly prescribed as shown in previous PPS and non-PPS studies.^47,60,61,64,82,93,95,116,125,126^ Our study revealed that other antibiotics that were highly used in the surveyed hospitals included benzylpenicillin, MTZ and amoxicillin, all from the Access group of antibiotics. This is consistent with a Global PPS across 53 countries which found that cephalosporins and penicillins are widely used globally.^32^ A study conducted in Tanzania also reported that ceftriaxone, metronidazole and amoxicillin were highly prescribed to inpatients.^99^ A Nigerian study also revealed that ceftriaxone and metronidazole were highly prescribed to hospitalized patients.^91^ Other studies have reported that cephalosporins, penicillins and metronidazole were highly prescribed to hospitalized patients.^97^ The high ceftriaxone usage reported here could be contributing to the rising of 3GC resistance in Zambia. A recent report at a large referral hospital indicated 3GC resistance at 54.9% in *Escherichia coli* and 73.9% in *Klebsiella pneumoniae*, suggesting that drugs like ceftriaxone may not only be ineffective but may worsen the problem.^127^ The overuse of ceftriaxone in hospitals could be due to its availability, wider coverage of pathogens, ease of administration and non-adherence to treatment guidelines.

In this study, the most diagnoses for antibiotic use included obstetrics and gynaecological infections, pneumonia and gastrointestinal tract infections. A study done in Nigeria also reported that pneumonia is among the leading contributors to the use of antibiotics in hospitalized patients.^128^ Additionally, most inpatients received antibiotics for surgical and medical prophylaxis and to treat CAIs. The high use of antibiotics for surgical and medical has been reported in other studies.^77,125^ Similarly, the overuse of antibiotics to treat CAIs has been reported in other studies.^96,129,130^ A study in Botswana also found that CAIs were the most diagnosed among 711 inpatients.^84^ A study in Saudi Arabia found that most patients received antibiotics to treat CAIs.^131^ Therefore, our study conforms to other studies that have reported that antibiotics are highly prescribed for inpatients suffering from CAIs and for surgical and medical prophylaxis.

Our study established that the compliance with the Zambian STG was 53%. The lowest compliance with the Zambian STGs was 0%, reported from two hospitals, while the highest was 57%, reported from two hospitals. Our study found a higher compliance to the STGs, unlike a previous PPS that reported a compliance of 27%.^64^ Another PPS conducted in Uganda found 30% compliance with the Ugandan STGs.^47^ A PPS in Tanzania found that 67.3% of the prescriptions complied with the Tanzanian STGs.^36^ In Canada, a PPS found that compliance with the treatment guidelines was 72%.^132^ In Zambia, further studies will be required to determine the reasons for the reported low compliance to STGs as adherence to the treatment guidelines promotes rational use of antibiotics and reduces the emergence and spread of AMR.

In this study, our findings demonstrate the need to instigate strategies to promote the rational use of antibiotics. We believe that establishing and strengthening AMS programmes in hospitals across Zambia would help to promote the rational use of antibiotics and reduce the emergence and spread of AMR. This is based on evidence on the impacts of AMS interventions on promoting the rational use of antibiotics.^28,133,134^

This study had some limitations. The PPS was conducted in 16 hospitals; hence, the findings cannot be generalized to all hospitals in Zambia. However, the findings derived from this study are valuable for developing strategies to promote rational prescribing and use of antibiotics in Zambia, given that the study was the largest PPS undertaken in the country. Hence, there is a need to instigate AMS programmes across hospitals in Zambia to promote rational prescribing and use of antibiotics, improve patient outcomes and reduce the emergence and spread of AMR.^18,22,28,29,135–140,141–143^ In resource-limited countries like Zambia, promoting infection prevention and control (IPC), hygiene, sanitation and AMS programmes at the institutional level is critical in addressing AMR.^144^ Additionally, there is a need to improve access to healthcare, promote rational use of antimicrobials and strengthen surveillance, provide adequate quality antimicrobials and foster global collaborations to combat AMR.^145^

Regarding policy implications, the health authorities in Zambia should ensure the availability of updated treatment guidelines in all healthcare facilities. Further, the health authorities must ensure that Access group antibiotics and treatment guidelines are readily available in all healthcare facilities to reduce the overuse of Watch group antibiotics. Furthermore, there is a need to establish and implement AMS programmes across all hospitals in the country and provide continuous professional development to healthcare workers on AMU, AMR and AMS. The healthcare authorities should promote annual or bi-annual PPS in hospitals to monitor and promote the rational use of antibiotics in Zambia.

### Conclusion

This study found a high prescription rate of antibiotics to inpatients across the 16 hospitals in Zambia. The high use of Watch group antibiotics indicated non-adherence to the WHO AWaRe classification of antibiotics and the National STGs. Therefore, there is a need to establish and strengthen AMS programmes in hospitals across Zambia.

## Transparency declarations

The authors have no relevant conflicts of interest to declare. All the authors do not have any financial interests or connections that may directly or indirectly raise concerns of bias in the work reported or the conclusions, implications or opinions made in this publication. The authors declared no potential conflicts of interest concerning the research, authorship and/or publication of this article. The authors declare that the research was conducted in the absence of any commercial or financial relationships that could be construed as a potential conflict of interest.
